# Loss of chymotrypsin-like protease (CTRL) alters intrapancreatic protease activation but not pancreatitis severity in mice

**DOI:** 10.1038/s41598-020-68616-9

**Published:** 2020-07-16

**Authors:** Dóra Mosztbacher, Zsanett Jancsó, Miklós Sahin-Tóth

**Affiliations:** 1 Center for Exocrine Disorders, Department of Molecular and Cell Biology, Boston University, Henry M. Goldman School of Dental Medicine, Boston, MA 02118 USA; 2 Department of Surgery, University of California Los Angeles, 675 Charles E Young Drive South, MacDonald Research Laboratories, Rm 2220, Los Angeles, CA 90095 USA

**Keywords:** Pancreatic disease, Pancreatitis, Acute pancreatitis

## Abstract

The digestive enzyme chymotrypsin protects the pancreas against pancreatitis by reducing harmful trypsin activity. Genetic deficiency in chymotrypsin increases pancreatitis risk in humans and pancreatitis severity in mice. Pancreatic chymotrypsin is produced in multiple isoforms including chymotrypsin B1, B2, C and chymotrypsin-like protease (CTRL). Here we investigated the role of CTRL in cerulein-induced pancreatitis in mice. Biochemical experiments with recombinant mouse enzymes demonstrated that CTRL cleaved trypsinogens and suppressed trypsin activation. We generated a novel CTRL-deficient strain (*Ctrl-KO*) using CRISPR-Cas9 genome engineering. Homozygous *Ctrl-KO* mice expressed no detectable CTRL protein in the pancreas. Remarkably, the total chymotrypsinogen content in *Ctrl-KO* mice was barely reduced indicating that CTRL is a low-abundance isoform. When given cerulein, *Ctrl-KO* mice exhibited lower intrapancreatic chymotrypsin activation and a trend for higher trypsin activation, compared with C57BL/6N mice. Despite the altered protease activation, severity of cerulein-induced acute pancreatitis was similar in *Ctrl-KO* and C57BL/6N mice. We conclude that CTRL is a minor chymotrypsin isoform that plays no significant role in cerulein-induced pancreatitis in mice.

## Introduction

The exocrine pancreas produces digestive proteases, each in several isoforms, that hydrolyze dietary proteins and peptides with distinct substrate specificities^1^. Trypsins cleave peptide chains after basic amino acids (Lys, Arg), chymotrypsins (CTRs) after aromatic (Tyr, Phe, Trp) and aliphatic (Leu, Met) amino acids while chymotrypsin-like elastases (CELAs) prefer to cleave after amino acids with aliphatic and/or small side chains (Ala, Val, Leu, Ile, Ser)^2^. Assignment of proteases to the chymotrypsin or elastase families is sometimes ambiguous as exemplified by CELA2A, which digests elastin but exhibits chymotrypsin-like substrate specificity. In contrast, human CELA3A and CELA3B display elastase-like specificity but digest elastin poorly, in all likelihood due to their acidic character. The C-terminal amino acids exposed by these digestive proteases are hydrolyzed by digestive carboxypeptidases (CPs). CPA1 and CPA2 cleave off aromatic and aliphatic amino acids from proteins and peptides while CPB1 releases basic amino acids.

In addition to their physiological function, digestive proteases also play important roles in the development of acute and chronic pancreatitis; the inflammatory disorders of the pancreas. Normally proteases are secreted by the pancreas as inactive precursors (trypsinogens, chymotrypsinogens, proelastases and procarboxypeptidases) and attain their active forms in the small intestine^3^. Premature, intrapancreatic activation of trypsin can initiate acute pancreatitis and drive its progression to chronic pancreatitis^4–6^. Remarkably, chymotrypsins are protective against pancreatitis due to their ability to degrade trypsinogens and thereby limit harmful trypsin activation^6,7^.

The human pancreas secretes 4 isoforms of chymotrypsinogens, CTRB1, CTRB2, CTRC and chymotrypsin-like protease (CTRL). All 4 isoenzymes exhibit characteristic chymotrypsin activity and hydrolyze polypeptides at Tyr and Phe residues^8^. CTRC also readily cleaves after Leu and Met residues while CTRB1 exhibits Trp preference as well. These differences in substrate recognition are due to the amino acids at positions 216 and 226 (crystallographic chymotrypsin numbering), which shape the mouth and the bottom of the substrate binding pocket, respectively. Human genetic studies revealed that loss-of-function mutations in CTRC impair protective trypsinogen degradation and increase pancreatitis risk significantly^6,7^. Furthermore, a frequently occurring inversion at the CTRB1-CTRB2 locus protects against pancreatitis by increasing CTRB2 expression resulting in more efficient trypsinogen degradation^9^. The role of CTRL in pancreatitis, however, has remained unclear.

A new mouse chymotrypsin isoform, named CTRA-1, was reported in 1991, and based on its strongly basic character this appears to be mouse CTRL^10^. The genomic sequence of human CTRL was first described in 1993, as one of 5 unrelated genes clustered on chromosome 16q22.1^11^. Subsequently, CTRL was purified and partially characterized in 1997 and renamed CTRL-1^12^. The rat ortholog was cloned and named chymopasin in 2002^13^. The human CTRL precursor protein is 26 kDa in size, basic in character (pI 8.6) and shares 40–56% amino-acid sequence identity with other chymotrypsin isoforms. The mouse ortholog shares 85% identity with the human enzyme. Using recombinant human CTRL, we determined the catalytic parameters on a series of p-nitroanilide peptide substrates and confirmed that CTRL was a chymotrypsin with a clear preference for P1 Tyr and Phe residues^8^. Nonetheless, phage-display selected CTRC inhibitors with a P1 Leu strongly inhibited human CTRL with nanomolar *K*_D_ values^14^.

In the present study, we set out to determine the significance of CTRL in experimental pancreatitis of mice. To this end, we generated a novel CTRL-deficient mouse strain (*Ctrl-KO*) and evaluated intrapancreatic protease activation and the severity of pancreatitis caused by cerulein injections. These studies were the logical extension of our recent reports that described the protective role of CTRB1 and CTRC in mice against cerulein-induced pancreatitis^15,16^.

## Materials and methods

### Accession numbers

NC_000074.6, *Mus musculus* strain C57BL/6J chromosome 8; NM_023182.2, *Mus musculus* chymotrypsin-like (*Ctrl*) mRNA.

### Expression plasmids

Construction of the pTrapT7-T7 and pTrapT7-T8 bacterial expression plasmids containing mouse cationic and anionic trypsinogen, respectively, were described previously^17^. The pcDNA3.1(-) mCTRL 10His and pcDNA3.1(-) mCTRB1 10His plasmids containing the mouse chymotrypsin-like protease and chymotrypsin B1 coding sequences with C-terminal 10His tags were constructed by custom synthesis of the genes, which were cloned into the vector with the XhoI and BamHI restriction sites.

### Expression and purification of mouse proteases

Mouse trypsinogens (isoforms T7 and T8) were expressed in *E. coli* and purified by ecotin affinity chromatography as described previously^17,18^. Concentration of trypsinogens was estimated from the UV absorbance at 280 nm^17^. Mouse CTRL and CTRB1 were expressed in HEK 293 T cells with transient transfection and purified from the conditioned medium by nickel affinity chromatography according to our published protocol^19^. Chymotrypsinogens were dialyzed against 0.1 M Tris–HCl (pH 8.0), 150 mM NaCl, and activated with trypsin beads (catalog number 20230, Thermo Fisher Scientific, Waltham, MA). The beads were removed by centrifugation and active CTRL and CTRB1 concentrations were determined by titration with ecotin^14^.

### Trypsinogen autoactivation assay

Measurement of trypsinogen autoactivation was performed as described previously^20^. Briefly, T7 and T8 mouse trypsinogens were incubated at 2 µM concentration with 10 nM initial trypsin in 0.1 M Tris–HCl (pH 8.0), 1 mM CaCl_2_ and 0.05% Tween 20 (final concentrations) at 37 °C in the absence or presence of 200 nM mouse CTRL or CTRB1. At the indicated times, 1.5 µL aliquots were withdrawn, mixed with 48.5 µL assay buffer (0.1 M Tris–HCl (pH 8.0), 1 mM CaCl_2_, 0.05% Tween 20) and trypsin activity was measured by adding 150 µL of 200 µM N-CBZ-Gly-Pro-Arg-*p*-nitroanilide substrate (catalog number L-1560, Bachem, Torrance, CA) dissolved in assay buffer. Trypsin activity was expressed as percent of the potential maximal activity, which was determined after full activation in the presence of 10 mM CaCl_2_.

### Animal studies approval

Description of the institutional approval and animal care was reported previously^16,20^. Specifically, animal experiments were performed at Boston University with the approval and oversight of the Institutional Animal Care and Use Committee (IACUC), including protocol review and post-approval monitoring. The animal care program at Boston University is managed in full compliance with the US Animal Welfare Act, the United States Department of Agriculture Animal Welfare Regulations, the US Public Health Service Policy on Humane Care and Use of Laboratory Animals and the National Research Council's Guide for the Care and Use of Laboratory Animals. Boston University has an approved Animal Welfare Assurance statement (A3316-01) on file with the US Public Health Service, National Institutes of Health, Office of Laboratory Animal Welfare and it is accredited by the Association for Assessment and Accreditation of Laboratory Animal Care International (AAALAC).

### Generation of the *Ctrl-KO* mouse strain

Mice were on the C57BL/6N genetic background. The gene encoding mouse CTRL is located on chromosome 8; it spans ~ 1.8 kb and comprises 7 exons (Supplementary Fig. S1). Mice deficient in CTRL (*Ctrl-KO*) were generated by CRISPR/Cas9-mediated genome engineering (Cyagen US Inc., Santa Clara, CA). Two guide RNAs were designed to target exon 3 of the *Ctrl* gene and in vitro transcribed Cas9 mRNA and the guide RNAs were injected into fertilized eggs. F0 founders were genotyped by PCR followed by DNA sequencing and positive mice were used for further breeding to generate F1 founders. One of 6 heterozygous F1 *Ctrl-KO* lines was backcrossed once with C57BL/6N mice and then bred to homozygosity and maintained in the homozygous state. C57BL/6N mice obtained from Charles River Laboratories (Wilmington, MA) or produced in our breeding facility from the same stock were used as experimental controls. The number of animals used in each experiment is shown in the figures. Both male and female mice were studied. Experimental mice were 10–12 weeks old and weighed around 25 g (males) and 20 g (females).

**Figure 1:**
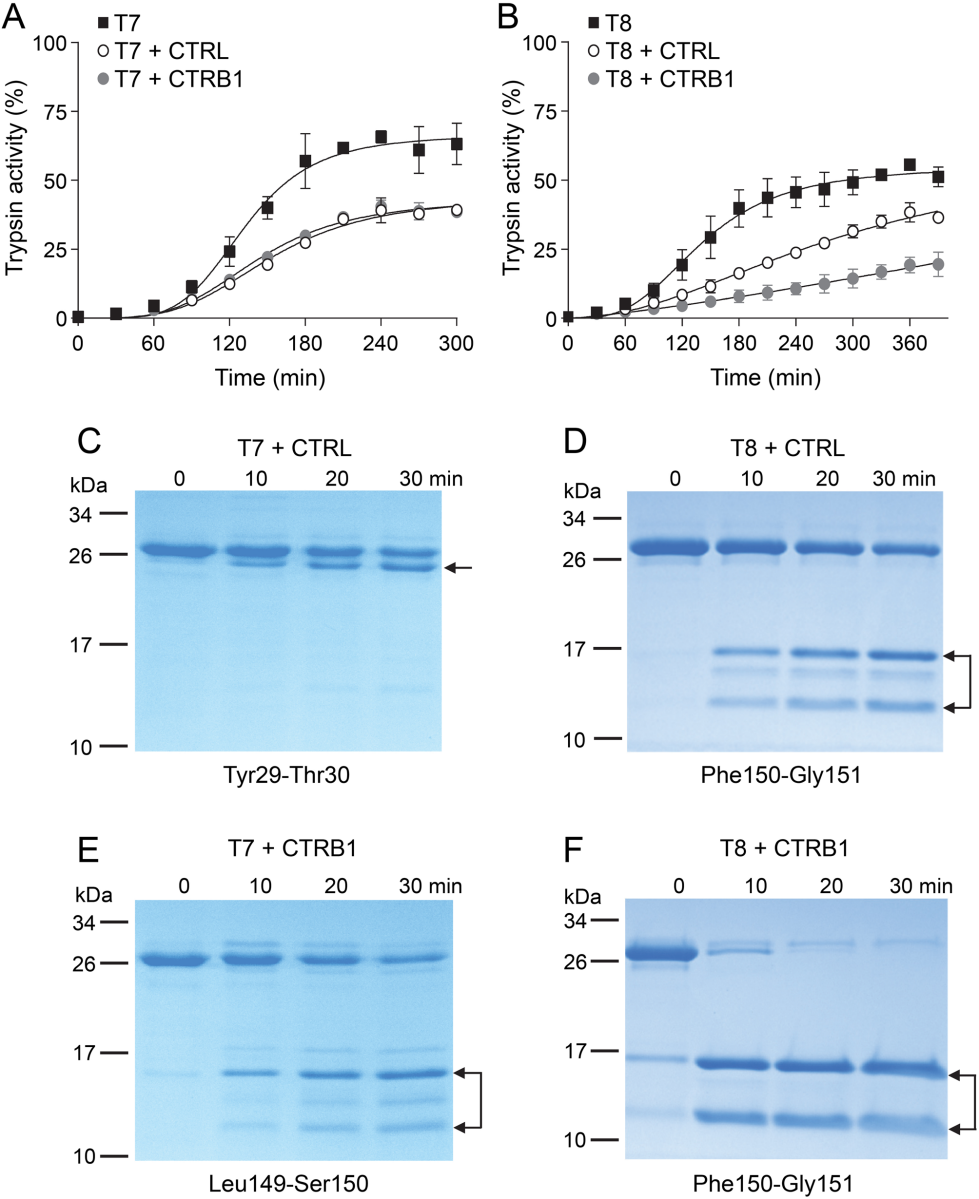
Effect of CTRL and CTRB1 on the autoactivation and proteolytic cleavage of mouse cationic (T7) and anionic (T8) trypsinogens. (**A**,**B**) Autoactivation of T7 and T8 mouse trypsinogens (2 µM) was measured in the absence or presence of 200 nM mouse CTRL or CTRB1, as described in “Methods”. Data points represent mean ± standard deviation (n = 2). (**C**–**F**) To study cleavage patterns, T7 and T8 mouse trypsinogens were incubated at 2 µM concentration in 0.1 M Tris–HCl (pH 8.0), at 37 °C with 200 nM mouse CTRL or CTRB1. At the indicated times, 100 µL aliquots were withdrawn, precipitated with 10% trichloroacetic acid (final concentration) and analyzed by SDS-PAGE and Coomassie Blue staining. Cleavage fragments are indicated by arrows. The main cleavage sites were determined by N-terminal sequencing of the fragments and are indicated below the gels. The CTRL cleavage site in T8 was predicted from the characteristic fragments.

### Genotyping

To genotype *Ctrl-KO* mice, we used primers that amplified exon 3 with flanking sequences (Supplementary Fig. S1). The amplicon size from C57BL/6N mice was 463 bp, whereas the deletion allele from *Ctrl-KO* mice yielded a 420 bp product. Forward primer: 5′-GAA GGC ATT AGG GAG AGG TAG ACT GG-3′, Reverse primer: 5′-GGT TCA GCA TTG GAA GAT CGG TC-3′.

### Reverse transcription (RT)-PCR

Expression levels of *Ctrl* and *Ctrb1* mRNA were determined by quantitative RT-PCR. Pancreas tissue (~ 30 mg) was freshly collected in RNA*later* (catalog number AM7020, Thermo Fisher Scientific) and total RNA was extracted using the RNeasy Plus Mini Kit (catalog number 74136, Qiagen, Valencia, CA). RNA (2 µg) was reverse-transcribed using the High Capacity cDNA Reverse Transcription Kit (catalog number 4368814, Thermo Fisher Scientific). Real-time PCR was performed using the TaqMan assays for mouse *Ctrl* (Mm00517786_m1), mouse *Ctrb1* (Mm00481616_m1) and the reference gene *Rpl13a* (mouse ribosomal protein L13a) (Mm01612987_g1). Relative expression levels were estimated with the comparative cycle threshold method (ΔΔCT method). First, CT values for the target gene were normalized to those of the *Rpl13a* reference gene (ΔCT) and then to the average ΔCT value of the C57BL/6N samples (ΔΔCT). Results were expressed as fold change calculated with the formula 2^−ΔΔCT^.

For qualitative analysis on agarose gels, the *Ctrl* coding region was amplified from pancreatic cDNA preparations using primers *Ctrl* cDNA F: 5′-GTC ACA ATG CTA CTG CTC AGC-3′ and *Ctrl* cDNA R: 5′-GGA ACG GAT CTG TGG ACA G-3′ (Supplementary Fig. S2). The product size from C57BL/6N mice was 821 bp while the predicted product size from the deletion allele of the *Ctrl-KO* mice was 778 bp. The 18S ribosomal RNA was amplified as housekeeping control gene using forward primer 5′-GAA ACG GCT ACC ACA TCC AAG G-3′ and reverse primer 5′-CCG CTC CCA AGA TCC AAC TAC G-3′. The amplicon size was 252 bp.

**Figure 2:**
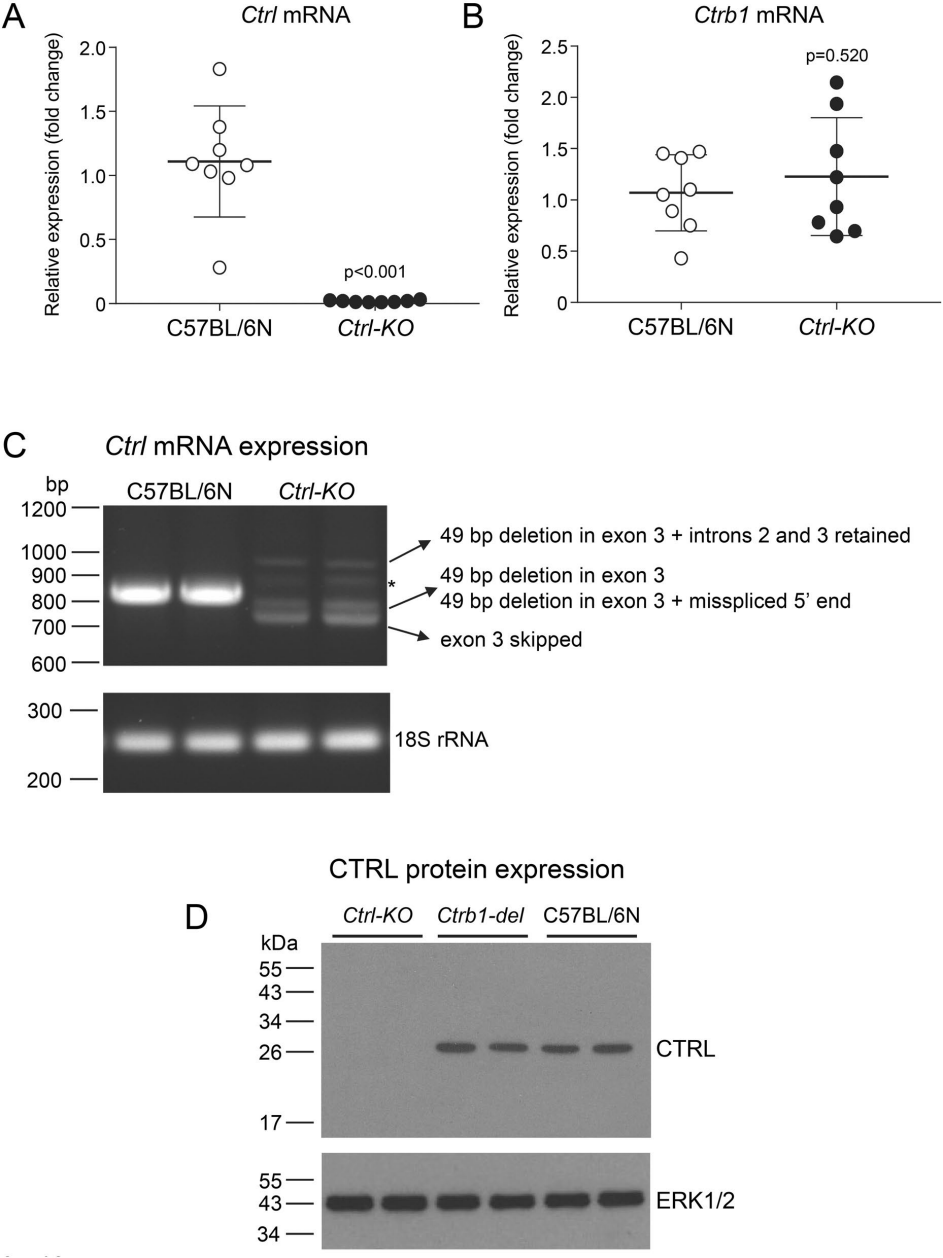
Expression of *Ctrl* mRNA and CTRL protein in the pancreas of *Ctrl-KO* mice. (**A**,**B**) Quantitative reverse-transcription PCR assay of pancreatic *Ctrl* (**A**) and *Ctrb1* (**B**) mRNA levels in C57BL/6N and *Ctrl-KO* mice. Individual data points with mean (horizontal bar) and standard deviation are shown. (**C**) Agarose gel analysis of reverse-transcription PCR of pancreatic *Ctrl* mRNA in C57BL/6N and *Ctrl-KO* mice. Note the multiple faint bands, which represent the indicated misspliced mRNA species in the *Ctrl-KO* mice. The band marked by the asterisk contains heteroduplexes. (**D**) Detection of CTRL protein in pancreas homogenates (30 µg total protein per lane loaded) from *Ctrl-KO*, *Ctrb1-del* and C57BL/6N mice by western blotting. See “Methods” for experimental details.

### Western blotting

Pancreas tissue (30 mg) was homogenized in 300 µL phosphate-buffered saline (pH 7.4) containing Halt Protease and Phosphatase Inhibitor Cocktail (catalog number 78440, Thermo Fisher Scientific) and 30 µg total protein of the cleared lysate was loaded per well. Mouse CTRL was detected using a rabbit polyclonal antibody raised against a synthetic peptide that corresponds to amino-acids 66–115 of human CTRL (catalog number AV33864, MilliporeSigma, St. Louis, MO). This sequence is 84% identical to the corresponding mouse CTRL region. The antibody was used at a final dilution of 1:1,000. Rabbit monoclonal antibody against p44/42 MAPK (ERK1/2) (137F5) was used at a final dilution of 1:500 (catalog number 4695, Cell Signaling Technology, Danvers, MA). The horseradish peroxidase-conjugated goat anti-rabbit secondary antibody was used at a dilution of 1:20,000 (catalog number 31460, Thermo Fisher Scientific).

### Pancreatic trypsinogen and chymotrypsinogen content

To determine the total pancreatic protease zymogen content, the pancreas tissue (30 mg) was homogenized in 300 μL 20 mM Na-HEPES (pH 7.4), and the homogenate was cleared by centrifugation (850 g, 10 min, 4 °C). Trypsinogen and chymotrypsinogen levels in the pancreas were measured enzymatically after maximal activation, as described recently^16,21^.

### Cerulein-induced pancreatitis

Acute pancreatitis was induced by repeated (10 times hourly) intraperitoneal injections of supramaximal stimulatory doses (50 µg/kg) of the secretagogue peptide cerulein (catalog number C9026, MilliporeSigma) dissolved in normal saline at 10 µg/mL. Control mice were given 10 hourly injections of normal saline. Mice were sacrificed 1 h after the last injection and blood and the pancreas were harvested.

### Pancreatic water content

A portion of the pancreas (50–100 mg) was weighed (wet mass), desiccated for 72 h at 65 °C and weighed again (dry mass). Pancreatic water content was calculated by subtracting the dry mass from the wet mass and it was expressed as percent of the wet mass.

### Plasma amylase

Blood was collected by cardiac puncture in heparinized syringes and cellular elements were removed by centrifugation (2,000 g, 10 min, 4 °C). The plasma was stored frozen at − 20 °C until use. Levels of amylase in blood plasma (1 µL assayed) were measured using the 2-chloro-*p*-nitrophenyl-α-D-maltotrioside substrate (catalog number A7564-60, Pointe Scientific, Canton, MI), as described previously^16,20^. Amylase activity was expressed in mOD/min units, 1 mOD/min corresponds to 23.8 U/L.

### Pancreas myeloperoxidase (MPO) content

Determination of the pancreas MPO content was carried out as reported previously^16,20^. Briefly, pancreas tissue was flash frozen in liquid nitrogen and stored at − 80 °C until use. Pancreas (25–30 mg) was homogenized in 800 µL of 10 mM Tris–HCl (pH 7.4), 200 mM NaCl, 5 mM K_2_-EDTA and 10% glycerol containing Halt Protease and Phosphatase Inhibitor Cocktail and MPO levels were measured with an ELISA kit (catalog number HK210-01, Hycult Biotech, Plymouth Meeting, PA), according to the manufacturer’s instructions. MPO concentrations were normalized to the total protein concentration and expressed in ng MPO/mg protein units.

### Pancreas histology

Microscopic analysis of the pancreas was performed as reported previously^16,20^. Briefly, pancreas tissue was fixed in 10% neutral buffered formalin; paraffin-embedded (FFPE); sectioned and stained with hematoxylin–eosin at the Boston University Experimental Pathology Laboratory Service Core. Arbitrary scoring (scale 0–3) by visual inspection was used to semi-quantitate the extent of tissue edema and inflammatory cell infiltration. 0 = absent, 1 = minimal (< 10% of visual field), 2 = moderate (10% to 50%), and 3 = severe (> 50%). Acinar cell necrosis was estimated by visual inspection and expressed as percent of tissue area.

### Intrapancreatic trypsin and chymotrypsin activation

Cerulein-induced intrapancreatic protease activation was determined at 30 min after a single cerulein (50 µg/kg) injection, using fluorescent substrates according to an optimized protocol reported recently^22^. Control mice were given saline injections. Protease activities were normalized to the total protein concentration in the assay and expressed in RFU/s/mg protein units.

### Statistical analysis

Results were graphed as individual data points with the mean and standard deviation indicated. Differences of means between two groups were analyzed by unpaired *t*-test. *P* < 0.05 was considered statistically significant.

## Results

### Mouse CTRL inactivates mouse trypsinogens T7 and T8

Chymotrypsins typically protect against pancreatitis by degrading trypsinogens. To establish whether mouse CTRL can cleave and thereby inactivate mouse trypsinogens, we followed autoactivation of mouse cationic and anionic trypsinogens (isoforms T7 and T8) in the absence and presence of 200 nM CTRL. Since mouse CTRB1 was previously shown to cleave T7 and T8 trypsinogens^15,17^, we compared the effect of CTRL to that of CTRB1. Both chymotrypsins decreased the final trypsin activity that developed as a result of trypsinogen autoactivation (Fig. 1A,B). While their impact was similar on T7 trypsinogen, CTRL was less effective on the T8 isoform than CTRB1. SDS-PAGE analysis revealed that CTRL cleaved the two trypsinogens at different sites and N-terminal protein sequencing identified the positions as the Tyr29-Thr30 peptide bond in the N-terminal region of T7 trypsinogen and the Phe150-Gly151 peptide bond in the autolysis loop of T8 trypsinogen (Fig. 1C,D, Supplementary Fig. S3). As reported before, CTRB1 cleaved T7 and T8 trypsinogens primarily in the autolysis loop at the Leu149-Ser150 and Phe150-Gly151 peptide bonds, respectively (Fig. 1E,F, Supplementary Fig. S3). CTRB1 cleaved the Phe150-Gly151 peptide bond in T8 trypsinogen more rapidly than CTRL (cf. Fig. 1D,F), which explains its stronger effect on autoactivation suppression (Fig. 1B). Taken together, the observations demonstrate the biochemical basis of a potential protective effect of CTRL against pancreatitis. Next, we tested this hypothesis in vivo using CTRL-deficient mice.

**Figure 3:**
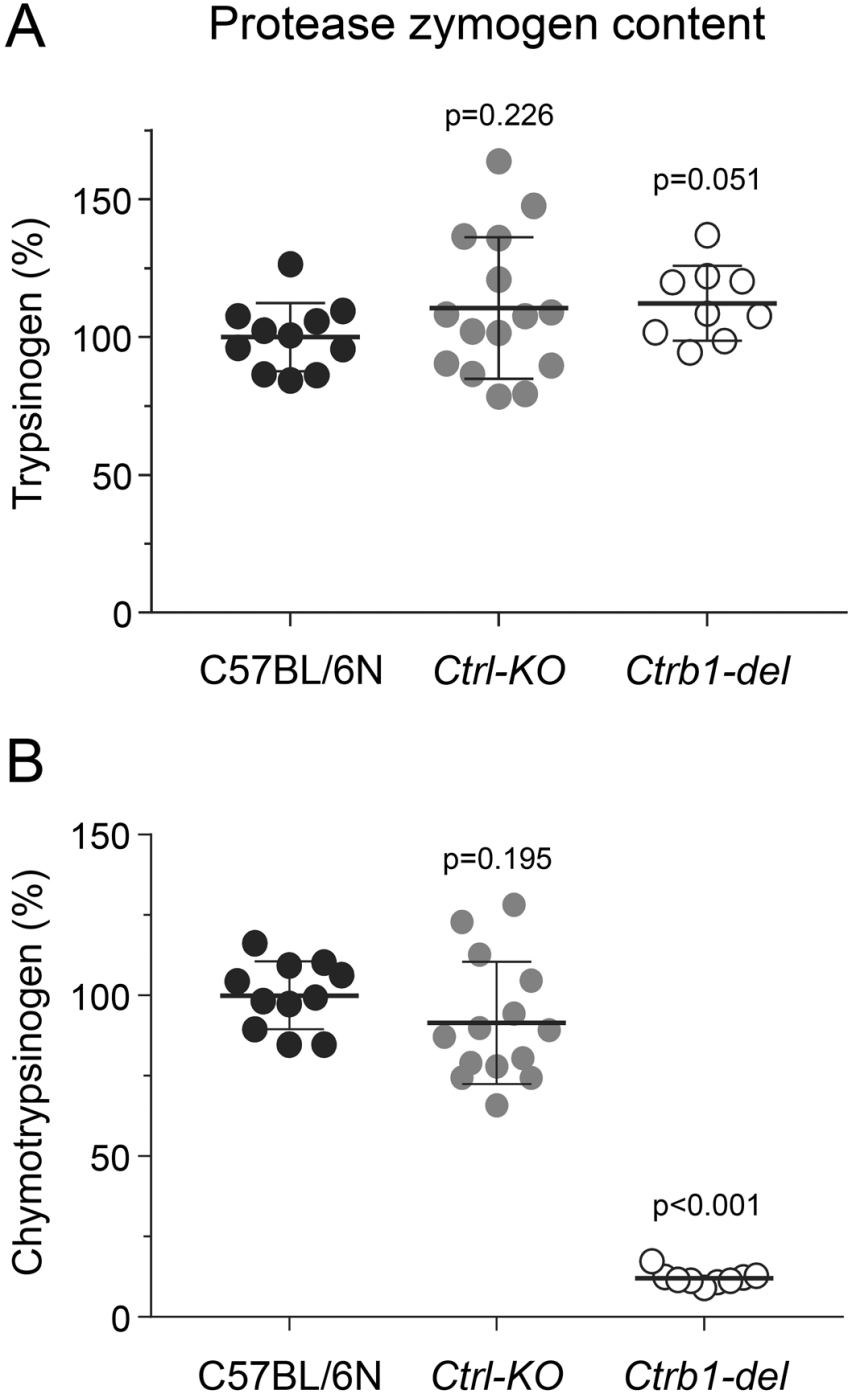
Protease zymogen content of the pancreas from *Ctrl-KO* mice. (**A**) Total trypsinogen and (**B**) chymotrypsinogen content was measured from pancreas homogenates of C57BL/6N, *Ctrl-KO* and *Ctrb1-del* mice, as detailed in “Methods”. Results were expressed as percent of the average C57BL/6N values. Individual data points with mean (horizontal bar) and standard deviation are shown.

### Generation of the *Ctrl-KO* mouse strain

We used CRISPR-Cas9 based genome editing in fertilized eggs of C57BL/6 mice to delete 43 bp from the beginning of exon 3 of the *Ctrl* gene (c.159_201del43). This deletion resulted in a frame shift and a premature termination codon downstream (p.D53EfsX52). See Supplementary Figs. S1 and S2 for *Ctrl* genomic and mRNA sequences and the position of the deletion. Mice were bred to homozygosity and were maintained in that state. Mice bred and developed normally, there was no phenotypic or behavioral difference from C57BL/6N controls. Pancreas morphology and histology was also unchanged (not shown).

### Protease expression in *Ctrl-KO* mice

We measured mRNA and protein levels of protease zymogens from pancreas homogenates of *Ctrl-KO* and C57BL/6N mice. For comparison, mice deficient in CTRB1 (*Ctrb1-del*) were also analyzed. The deletion in exon 3 of the *Ctrl* gene was predicted to cause nonsense-mediated mRNA decay. This was confirmed by quantitative reverse-transcription PCR, which showed almost no detectable *Ctrl* mRNA (Fig. 2A). In contrast, expression of *Ctrb1* mRNA was comparable in *Ctrl-KO* and C57BL/6N mice (Fig. 2B). When the *Ctrl* coding DNA was amplified from pancreatic cDNA and analyzed on agarose gels, a single strong band was obtained from C57BL/6N control mice, whereas multiple faint bands with abnormal migration were observed from *Ctrl-KO* mice (Fig. 2C, Supplementary Fig. S4). DNA sequencing of the spurious bands revealed that these were various misspliced forms of *Ctrl* mRNA as detailed in Fig. 2C.

**Figure 4:**
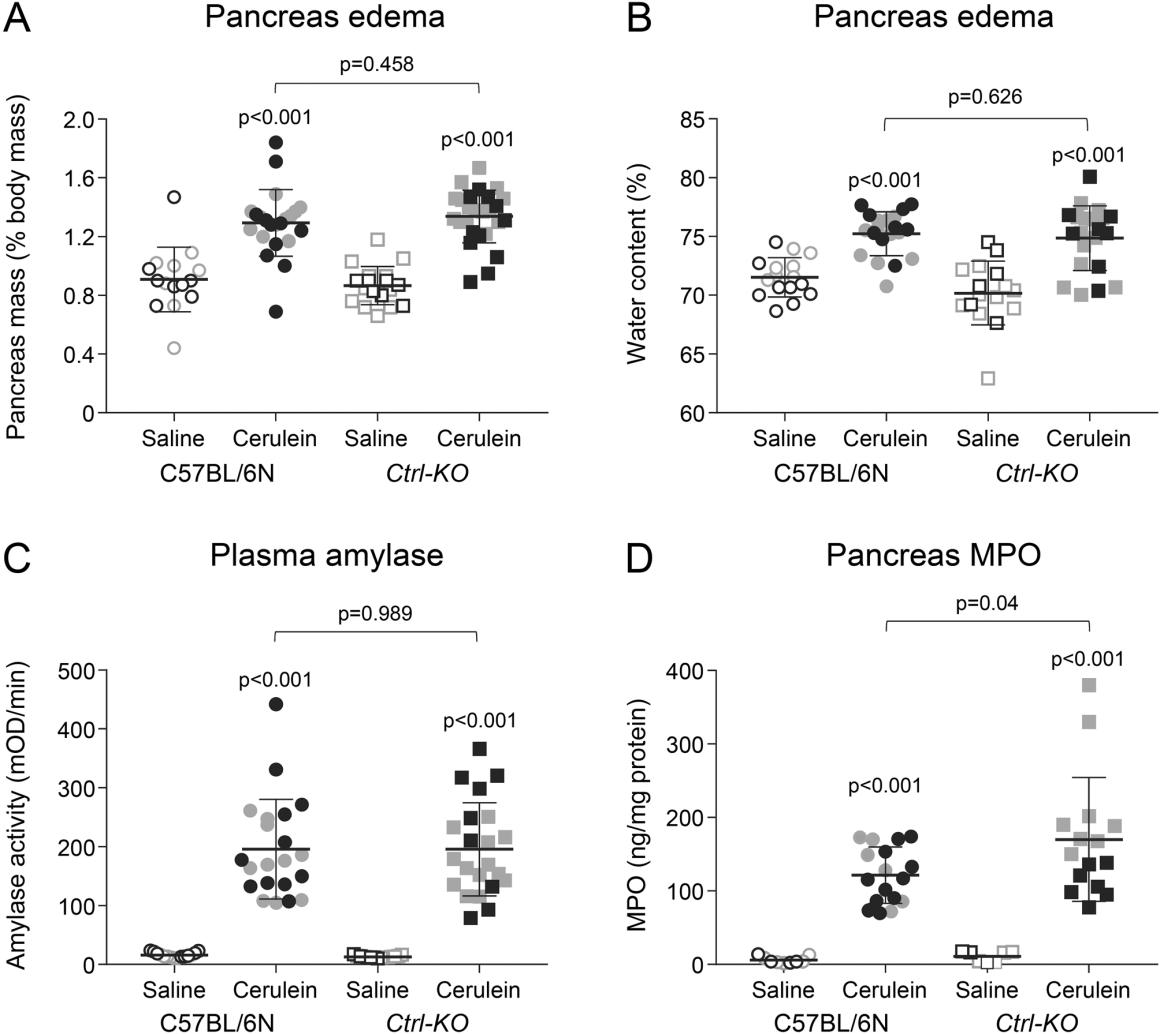
Cerulein-induced acute pancreatitis in *Ctrl-KO* mice. Parameters of pancreatitis severity in C57BL/6N and *Ctrl-KO* mice were determined after 10 hourly injections of cerulein or saline. (**A**) Pancreas mass, expressed as percent of body mass. (**B**) Pancreatic water content, expressed as percent of the wet pancreas mass. (**C**) Plasma amylase activity. (**D**) Pancreatic myeloperoxidase (MPO) content. Individual data points with mean (horizontal bar) and standard deviation are shown. Gray symbols indicate female mice. See “Methods” for experimental details.

Western blot analysis using a polyclonal antibody raised against the homologous human CTRL showed no detectable protein in pancreas extracts of *Ctrl-KO* mice, whereas a single band of the expected size was found in C57BL/6N controls and in *Ctrb1-del* mice (Fig. 2D, Supplementary Fig. S5). Total pancreatic trypsinogen content was comparable in *Ctrl-KO*, *Ctrb1-del* and C57BL/6N mice (Fig. 3A). Surprisingly, the total chymotrypsinogen content in the pancreas of *Ctrl-KO* mice was not significantly different from that of C57BL/6N controls; although a slight reduction (circa 10%) was evident as a trend (Fig. 3B). In contrast, as reported previously^15^, total chymotrypsinogen content in the pancreas of *Ctrb1-del* mice was markedly reduced with about 10% residual activity observed. Taken together, the measurements indicate that CTRL is a minor chymotrypsin isoform in mice, which constitutes about 10% of total chymotrypsinogens.

**Figure 5:**
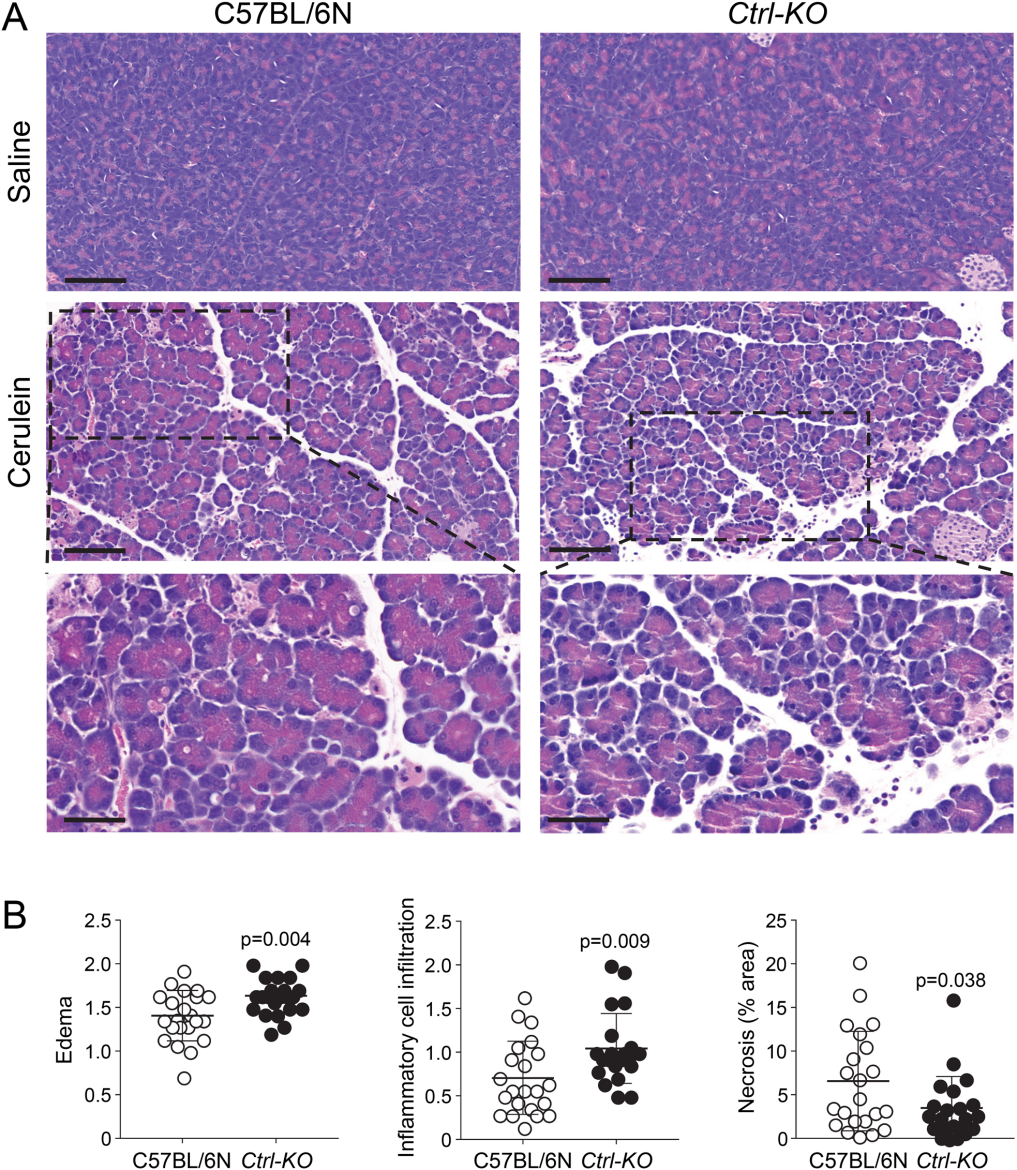
Histology of cerulein-induced acute pancreatitis in *Ctrl-KO* mice. (**A**) Representative hematoxylin–eosin stained histological sections of the pancreas from saline and cerulein-treated C57BL/6N and *Ctrl-KO* mice. Scale bars correspond to 100 µm (top two rows) or 50 µm (magnified regions, bottom row). (**B**) Histology scoring for edema, inflammatory cell infiltration, and acinar cell necrosis in cerulein-treated mice. Individual data points with mean (horizontal bar) and standard deviation are shown. See “Methods” for details.

### Cerulein-induced pancreatitis in *Ctrl-KO* mice

To investigate whether the potential protective effect of CTRL would alter severity of secretagogue hyperstimulation-induced pancreatitis, we compared pancreatitis responses in *Ctrl-KO* and C57BL/6N mice after 10 hourly injections of 50 µg/kg cerulein (Figs. 4, 5). We determined changes in pancreas mass (Fig. 4A), pancreatic water content (Fig. 4B), plasma amylase levels (Fig. 4C), and pancreas myeloperoxidase (MPO) content (Fig. 4D). All these parameters increased significantly in mice given cerulein versus mice given saline injections. However, with the exception of MPO content, we found no significant differences between *Ctrl-KO* and C57BL/6N mice. Pancreatic MPO content was somewhat higher in cerulein-treated *Ctrl-KO* versus C57BL/6N mice (Fig. 4D). Interestingly, this effect was due to the higher MPO levels seen in female mice while no gender difference was apparent in pancreatic mass, pancreatic water content and plasma amylase (Fig. 4).

We also analyzed pancreas sections by hematoxylin–eosin staining and histology scoring (Fig. 5). Visual inspection revealed highly similar microscopic pancreas morphology in cerulein-treated *Ctrl-KO* and C57BL/6N mice with characteristic edema, inflammatory cell infiltration and acinar cell necrosis (Fig. 5A). Scoring 22 cerulein-treated mice from each strain revealed small differences in edema, inflammatory cell infiltration and necrosis of the pancreas between *Ctrl-KO* mice and C57BL/6N controls (Fig. 5B). The histological increase in inflammatory cell infiltration in *Ctrl-KO* mice seemed consistent with the modest MPO elevation found in pancreas homogenates (Fig. 4D). The extent of acinar cell necrosis, as defined by intense pink areas with loss of cellular architecture, was low (around 5%), as we typically find under these experimental conditions. Although somewhat less acinar necrosis was evident in *Ctrl-KO* mice versus C57BL/6N controls, the difference seems biologically irrelevant. Apoptotic acinar cells with shrunken morphology and condensed nuclei were also evident, with comparable frequency in *Ctrl-KO* and C57BL/6N mice.

### Intrapancreatic protease activation in Ctrl-KO mice

**Figure 6:**
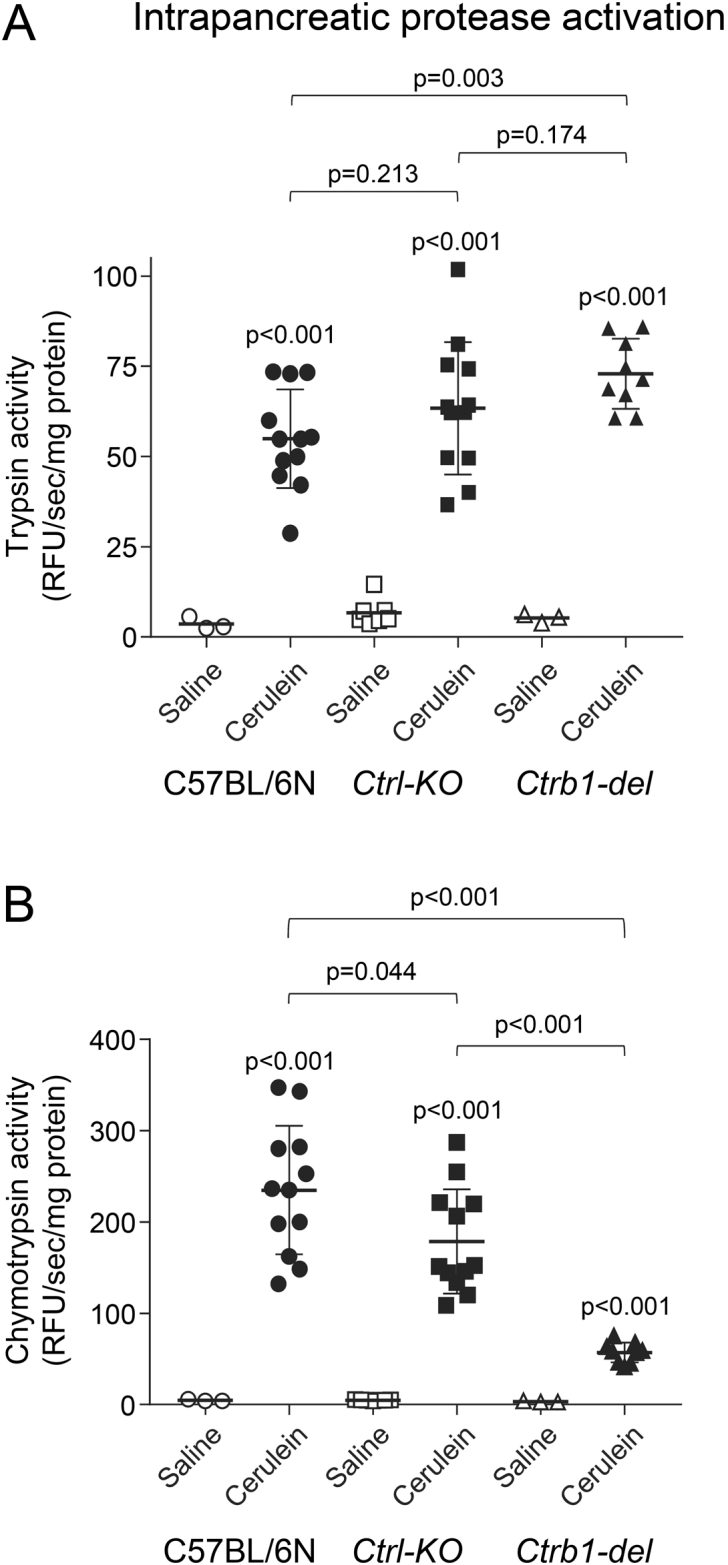
Intrapancreatic protease activation in *Ctrl-KO* mice. (**A**) Trypsin and (**B**) chymotrypsin activities were measured from pancreas homogenates of C57BL/6N, *Ctrl-KO* and *Ctrb1-del* mice 30 min after a single saline or cerulein injection. Individual data points with mean (horizontal bar) and standard deviation are shown. See “Methods” for experimental details.

To assess the extent of cerulein-induced intrapancreatic trypsin and chymotrypsin activation, we measured protease activities from pancreas homogenates of *Ctrl-KO* and C57BL/6N mice 30 min after a single cerulein injection. For comparison, *Ctrb1-del* mice were also included in this experiment. We recently optimized the methodology for measuring cerulein-induced trypsin and chymotrypsin activation from pancreas homogenates^22^. Here we report updated results for the intrapancreatic protease activation of *Ctrb1-del* mice, which are qualitatively similar to those in our prior publication^15^. Relative to saline-injected mice, there was a significant increase in intrapancreatic trypsin and chymotrypsin activity in all 3 strains given cerulein (Fig. 6). When compared to C57BL/6N mice, trypsin activity was about 15% higher in the pancreas of *Ctrl-KO* mice, although the difference did not reach statistical significance (Fig. 6A). In contrast, trypsin activation was significant and more robust (33% increase) in *Ctrb1-del* mice, as reported before^15^. Intrapancreatic chymotrypsin activation was significantly lower in both *Ctrl-KO* and *Ctrb1-del* mice versus C57BL/6N mice (Fig. 6B). However, the decrease in chymotrypsin activation was relatively modest (24%) in *Ctrl-KO* mice while a marked reduction (76%) was seen in *Ctrb1-del* mice, in agreement with previous observations^15^. Taken together, the results indicate that despite its low abundance, CTRL contributes to cerulein-induced intrapancreatic chymotrypsin activation to a measurable degree and loss of CTRL activity is associated with a small increase in intrapancreatic trypsin activation.

## Discussion

In the present study we investigated the role of CTRL in cerulein-induced pancreatitis in mice. Previously, we established that chymotrypsins exert a protective effect in this pancreatitis model by mitigating intrapancreatic trypsin activation through trypsinogen degradation. Thus, in *Ctrb1-del* mice, the genetic deletion of the most abundant chymotrypsin CTRB1 resulted in elevated intrapancreatic trypsin activation and more severe cerulein-induced pancreatitis^15^. C57BL/6N mice are naturally deficient in CTRC due to a single nucleotide deletion in exon 2 of the *Ctrc* gene. Restoration of a functional *Ctrc* gene in C57BL/6N mice caused reduced intrapancreatic trypsin activation and less severe pancreatitis^16^. In contrast to the demonstrated protective effects of CTRB1 and CTRC in mice against pancreatitis, the observations reported here did not indicate a similar role for CTRL. Biochemical experiments confirmed that CTRL is capable of digesting mouse cationic and anionic trypsinogens (isoforms T7 and T8). However, pancreatic expression levels of CTRL were low (10% of total chymotrypsinogens), which may limit its efficacy in mediating trypsinogen degradation. *Ctrl-KO* mice deficient in CTRL exhibited a nearly 25% reduction in cerulein-induced intrapancreatic chymotrypsin activation with a concomitant 15% increase in trypsin activation, indicating that CTRL contributes to protection against trypsin activation to a measurable, albeit small, extent. Still, the CTRL-dependent changes in intrapancreatic protease activation did not have a significant impact on the severity of cerulein-induced acute pancreatitis. With the exception of higher inflammatory cell infiltration in *Ctrl-KO* mice, parameters of severity studied were comparable in *Ctrl-KO* and C57BL/6N mice. A caveat to these results is that pancreatitis responses were analyzed only at a given time point (i.e. 1 h after the tenth cerulein injection) and it is possible that at later time points or with different cerulein administration protocols the studied outcomes might show differences between *Ctrl-KO* and C57BL/6N mice. Nonetheless, the present study clearly indicates that CTRL is a minor chymotrypsin isoform in mice that is relatively unimportant for pancreatitis. However, in humans, we do not know the relative contribution of CTRL to the pancreatic chymotrypsin levels and we cannot rule out that CTRL might play a more significant protective role than in mice.

The protective effect of chymotrypsins against pancreatitis was first discovered by human genetic studies that investigated association of *CTRC* mutations and chronic pancreatitis^6,7,23^. CTRC missense variants and a microdeletion were shown to cause loss of CTRC function by various mechanisms that included loss of secretion, impaired catalytic activity and increased sensitivity to degradation by trypsin^23–25^. Some CTRC mutants also caused endoplasmic reticulum stress when expressed in cell culture^24,26^. Overall, loss-of-function CTRC mutations were found in about 4–5% of patients and they increased pancreatitis risk by 5–tenfold^23,27^. Candidate gene and GWAS studies also revealed that the common CTRC variant c.180C > T (p.G60 =) increased pancreatitis risk about twofold in the heterozygous state^9,28^. Remarkably, in the homozygous state the variant was often found in pediatric cases where it increased risk by more than tenfold^29^. The p.G60 = variant likely reduces CTRC expression at the mRNA level, possibly through altered splicing, although experimental evidence is lacking.

The CTRB1 gene is duplicated in humans and a common inversion (allele frequency 18%) is found at the *CTRB1-CTRB2* locus, which switches the 5′ region and exon 1 between the two chymotrypsin genes. This results in altered expression levels of CTRB2, which can degrade trypsinogens more efficiently than CTRB1^9^. The overall impact of the inversion is a small protective effect (about 1.4-fold) against chronic pancreatitis. Even though CTRB1 and CTRB2 together are the most abundant chymotrypsins in humans, there are no reported mutations in these proteases in association with pancreatitis. A commonly occurring deletion variant of CTRB2 was described (allele frequency 7%), however, despite the obvious loss of function, no association with pancreatitis was documented to date^9^. Thus, relative to CTRC, the contribution of CTRB1 and CTRB2 to the chymotrypsin-dependent protection against pancreatitis in humans seems limited. The potential link of variants in the human *CTRL* gene and chronic pancreatitis has not been investigated so far. However, GWAS studies performed to date revealed no common variants in this gene that would alter pancreatitis risk^9^.

Human and mouse studies offered remarkably consistent evidence for the protective role of chymotrypsins against pancreatitis. While it is possible that chymotrypsins have multiple targets through which they prevent and/or mitigate pancreatitis; the only mechanism elucidated to date is proteolytic cleavage of trypsinogens, which in turn, limits intrapancreatic trypsin activation^6,7^. The notion that such a powerful protease as chymotrypsin does not harm the pancreas and even protects it, argues against the non-specific autodigestion theory of pancreatitis pathogenesis and strongly suggests that specific trypsin targets are responsible for disease onset.

In conclusion, genetic disruption of the *Ctrl* gene in mice caused a modest decrease in cerulein-induced intrapancreatic chymotrypsin activation, which was associated with slightly higher intrapancreatic trypsin activation. However, the small increase in trypsin activation did not translate to appreciable changes in pancreatitis severity in *Ctrl-KO* mice given cerulein. Therefore, CTRL does not play a significant role in secretagogue-induced pancreatitis in mice.

## Supplementary information

Supplementary file1 (PDF 1778 kb)

## Data Availability

Materials, data and protocols associated with this paper are available upon request.
