# Supplementary material for: Loss of chymotrypsin-like protease (CTRL) alters intrapancreatic protease activation but not pancreatitis severity in mice
Source: Sci Rep. 2020 Jul 16;10:11731. doi: 10.1038/s41598-020-68616-9 (PMC7366634; doi:10.1038/s41598-020-68616-9)

SUPPLEMENTARY MATERIAL

Loss of chymotrypsin-like protease (CTRL) alters intrapancreatic protease activation but not pancreatitis severity in mice

Dóra Mosztbacher[1], Zsanett Jancsó[1,2], Miklós Sahin-Tóth[1,2*]

[1]Center for Exocrine Disorders, Department of Molecular and Cell Biology, Boston University, Henry M. Goldman School of Dental Medicine, Boston, Massachusetts 02118 and [2]Department of Surgery, University of California Los Angeles, Los Angeles, California 90095

Supplementary Figure 1. Genomic sequence of mouse *Ctrl* (NC_000074.6). Exons are highlighted in yellow and the translation start and stop codons are in green. The 5' and 3' splice sites are italicized and underlined. The polyadenylation signal sequence in the 3' region is underlined and emboldened. The position of the primers used for genotyping are underlined and shown in bold typeface. The 43 bp deletion (c.159_201del43) in exon 3 of the *Ctrl-KO* mice is indicated in blue.

Genotyping primers:
mCtrl-F: 5'-GAA GGC ATT AGG GAG AGG TAG ACT GG-3'
mCtrl-R: 5'-GGT TCA GCA TTG GAA GAT CGG TC-3'
PCR product sizes: C57BL/6N 463 bp, *Ctrl-KO* 420 bp

GACCATCTCAACACCATTCCTTATTTGTCACA**ATG**CTACTGCTCAGCCTAACCCTTAGCCTGGTCCTCCTTGGCTCC
TCCTGGG*GTAAGT*GGGCTGGGAACCGACCTTGGCTGTTCAGGGGCCCAGACAAGGAGCCAGCCTGATGAGAAGGGAA
CGGACAGTGTAAGCAAAAGGAAGGAACCTTCAGGGCTGAGAGTAAGGGAAGAGCCAGGGACCGGTCTCACAAATGAG
GCGGGAGCTTGAGCCAGACCTAAGGAGGCTCAGGAACATATAAGAGATGTGTATAGACTAACAAAGCCTTCTAGCTG
CATGGTAGACTGTGCAGGCCTAGAAAAACAGAGGGCAGGGAGATGATAGCAGGAATTAGAGAGAAAACTGGCCCAAG
GTACATTAGGACTAGGTTGGTAGCTGTGAAAGCATGCACAGGACTTGAAGGCTGGTAGGGTGGGGTAACCAAGATAG
CTGTGTATGCCTAAAAGGCACTCAAGGGAAGGCCTTGGAGTAATTCTCTTGGACCTCCTCCTGGATCTACAGCCTGG
GGGCACCTTGAA**GAAGGCATTAGGGAGAGGTAGACTGG**GATTGGATTCTCACCAGCCCTATCTATGTT*CAG*GCTGTG
GTGTTCCTGCCATCACGCCTGCACTGAGCTACAATCAGAGAATTGTCAACGGGGAGAATGCAGTGCCAGGCTCCTGG
CCCTGGCAGGTGTCTCTCCAG*GTAATG*CACCCTATGGGTGGGTTGGGGTCCTGGGTGCTTTAGGCTATGGGTAACTT
AAGCCCATTCATGTATCTAACTTTTGACCC*TAG*GA**TAACACCGGCTTCCACTTCTGCGGTGGTTCTCTCATCAGTCC**
GAACTGGGTGGTCACGGCTGCCCACTGCCAAGTCAC*GTGAGT*GTATCCCCCTCCCTTGGATGCCCGCTCTGAAACCA
CTTAGCAGCTGCCCATTCCCATTATCCTTG*CAG*GCCTGGACGCCACTTTGTCGTTTTGGGAGAATAT**GACCGATCTT**
CCAATGCTGAACCTGTGCAGGTCCTCTCGATCGCAAGG*GTAAGT*ACTAGCTGTGCATGTAGAAAGGGAAAATGGACT
GCAGGGCATCCAGGCATGCCCTGACCTACCCGCCTCAGCACAGGCCTGGGCACCTGTGCCCACTCCAGCTTTACATG
ACTACTGACTGGCTCCA*CAG*GCCATCACACACCCTAACTGGAACGCCAACACCATGAACAATGACCTGACTCTCCTG
AAGCTTGCCTCGCCAGCCCGGTACACAGCACAAGTCTCACCAGTCTGCCTGGCTTCCACAAACGAGGCACTGCCTTC
GGGGCTCACCTGTGTCACCACTGGCTGGGGCCGAATCAGTGGTGTGG*GTAAGA*ACCTGGGTAATGAGGGGAGACCAG
AAGAGCACCATGGGCCAGCCTAACCACCTCTCCTGGCCCA*CAG*GCAATGTGACACCAGCTCGCCTGCAGCAAGTTGT
TCTACCCCTGGTCACTGTGAATCAGTGTCGGCAGTACTGGGGTGCACGCATTACCGATGCCATGATATGTGCAGGTG
GCTCAGGCGCCTCCTCATGTCAG*GTAAGC*ACCCTGTCCTCCGCACTGTCCAGTGGGACACGTCCCCAAACTGCCATG
CTGACTTCTCCCCTTTTCTTCCCTTT*CAG*GGTGACTCAGGAGGCCCTCTTGTCTGCCAGAAGGGAAACACCTGGGTG
CTTATTGGGATTGTCTCCTGGGGCACTAAGAACTGCAACATACAAGCACCGGCCATGTACACTCGGGTCAGCAAGTT
CAGTACCTGGATCAACCAAGTCATGGCCTACAAC**TAA**ACTGTCCACAGATCCGTTCCCCATCTCAATCT**AATAAA**CA
TACTCGTCTCAGTTTCTCTTG

Supplementary Figure 2. Messenger RNA sequence of *Ctrl* from C57BL/6N mice (NM_023182.2) and predicted mRNA sequence of *Ctrl* from *Ctrl-KO* mice. Amino-acid translations are also shown. The translation start and stop codons are indicated in green with underlined, bold typeface. The positions of primers used to amplify *Ctrl* cDNA are underlined and emboldened.

Primers used for cDNA amplification:
Ctrl cDNA F: 5′-GTC ACA ATG CTA CTG CTC AGC-3′
Ctrl cDNA R: 5′-GGA ACG GAT CTG TGG ACA G-3′
PCR product size: C57BL/6N mice, 821 bp

Mouse *Ctrl* mRNA

GACCATCTCAACACCATTCCTTATTT**GTCACAATGCTACTGCTCAGC**CTAACCCTTAGCCTGGTCCTCCTTGGCTCC
TCCTGGGGCTGTGGTGTTCCTGCCATCACGCCTGCACTGAGCTACAATCAGAGAATTGTCAACGGGGAGAATGCAGT
GCCAGGCTCCTGGCCCTGGCAGGTGTCTCTCCAGGATAACACCGGCTTCCACTTCTGCGGTGGTTCTCTCATCAGTC
CGAACTGGGTGGTCACGGCTGCCCACTGCCAAGTCACGCCTGGACGCCACTTTGTCGTTTTGGGAGAATATGACCGA
TCTTCCAATGCTGAACCTGTGCAGGTCCTCTCGATCGCAAGGGCCATCACACACCCTAACTGGAACGCCAACACCAT
GAACAATGACCTGACTCTCCTGAAGCTTGCCTCGCCAGCCCGGTACACAGCACAAGTCTCACCAGTCTGCCTGGCTT
CCACAAACGAGGCACTGCCTTCGGGGCTCACCTGTGTCACCACTGGCTGGGGCCGAATCAGTGGTGTGGGCAATGTG
ACACCAGCTCGCCTGCAGCAAGTTGTTCTACCCCTGGTCACTGTGAATCAGTGTCGGCAGTACTGGGGTGCACGCAT
TACCGATGCCATGATATGTGCAGGTGGCTCAGGCGCCTCCTCATGTCAGGGTGACTCAGGAGGCCCTCTTGTCTGCC
AGAAGGGAAACACCTGGGTGCTTATTGGGATTGTCTCCTGGGGCACTAAGAACTGCAACATACAAGCACCGGCCATG
TACACTCGGGTCAGCAAGTTCAGTACCTGGATCAACCAAGTCATGGCCTACAAC**TAA**A**CTGTCCACAGATCCGTTCC**
CCATCTCAATCT**AATAAA**CATACTCGTCTCAGTTTCTCTTG

Translation of mouse *Ctrl* coding DNA
Blue highlights the secretory signal peptide and yellow indicates the activation peptide.

MLLLSLTLSLVLLGSSWGCGVPAITPALSYNQRIVNGENAVPGSWPWQVSLQDNTGFHFCGGSLISPNWVVTAAHCQ
VTPGRHFVVLGEYDRSSNAEPVQVLSIARAITHPNWNANTMNNDLTLLKLASPARYTAQVSPVCLASTNEALPSGLT
CVTTGWGRISGVGNVTPARLQQVVLPLVTVNQCRQYWGARITDAMICAGGSGASSCQGDSGGPLVCQKGNTWVLIGI
VSWGTKNCNIQAPAMYTRVSKFSTWINQVMAYN**X**

Predicted mouse *Ctrl* mRNA with 43 bp deletion (c.159_201del43)

GACCATCTCAACACCATTCCTTATTT**GTCACAATGCTACTGCTCAGC**CTAACCCTTAGCCTGGTCCTCCTTGGCTCC
TCCTGGGGCTGTGGTGTTCCTGCCATCACGCCTGCACTGAGCTACAATCAGAGAATTGTCAACGGGGAGAATGCAGT
GCCAGGCTCCTGGCCCTGGCAGGTGTCTCTCCAGGAAACTGGGTGGTCACGGCTGCCCACTGCCAAGTCACGCCTGG
ACGCCACTTTGTCGTTTTGGGAGAATATGACCGATCTTCCAATGCTGAACCTGTGCAGGTCCTCTCGATCGCAAGGG
CCATCACACACCCTAACTGGAACGCCAACACCATGAACAATGACCTGACTCTCCTGAAGCTTGCCTCGCCAGCCCGG
TACACAGCACAAGTCTCACCAGTCTGCCTGGCTTCCACAAACGAGGCACTGCCTTCGGGGCTCACCTGTGTCACCAC
TGGCTGGGGCCGAATCAGTGGTGTGGGCAATGTGACACCAGCTCGCCTGCAGCAAGTTGTTCTACCCCTGGTCACTG
TGAATCAGTGTCGGCAGTACTGGGGTGCACGCATTACCGATGCCATGATATGTGCAGGTGGCTCAGGCGCCTCCTCA
TGTCAGGGTGACTCAGGAGGCCCTCTTGTCTGCCAGAAGGGAAACACCTGGGTGCTTATTGGGATTGTCTCCTGGGG
CACTAAGAACTGCAACATACAAGCACCGGCCATGTACACTCGGGTCAGCAAGTTCAGTACCTGGATCAACCAAGTCA
TGGCCTACAAC**TAA**A**CTGTCCACAGATCCGTTCC**CCATCTCAATCT**AATAAA**CATACTCGTCTCAGTTTCTCTTG

Translation of predicted mouse *Ctrl* coding DNA with deletion (p.D53EfsX52)
Blue highlights the secretory signal peptide and yellow indicates the activation peptide.
The frame-shifted sequence is shown in gray.

MLLLSLTLSLVLLGSSWGCGVPAITPALSYNQRIVNGENAVPGSWPWQVSLQETGWSRLPTAKSRLDATLSFWENMT
DLPMLNLCRSSRSQGPSHTLTGTPTP**X**

Supplementary Figure 3. Uncropped version of gels shown in Figure 1.

Figure 1 C and E

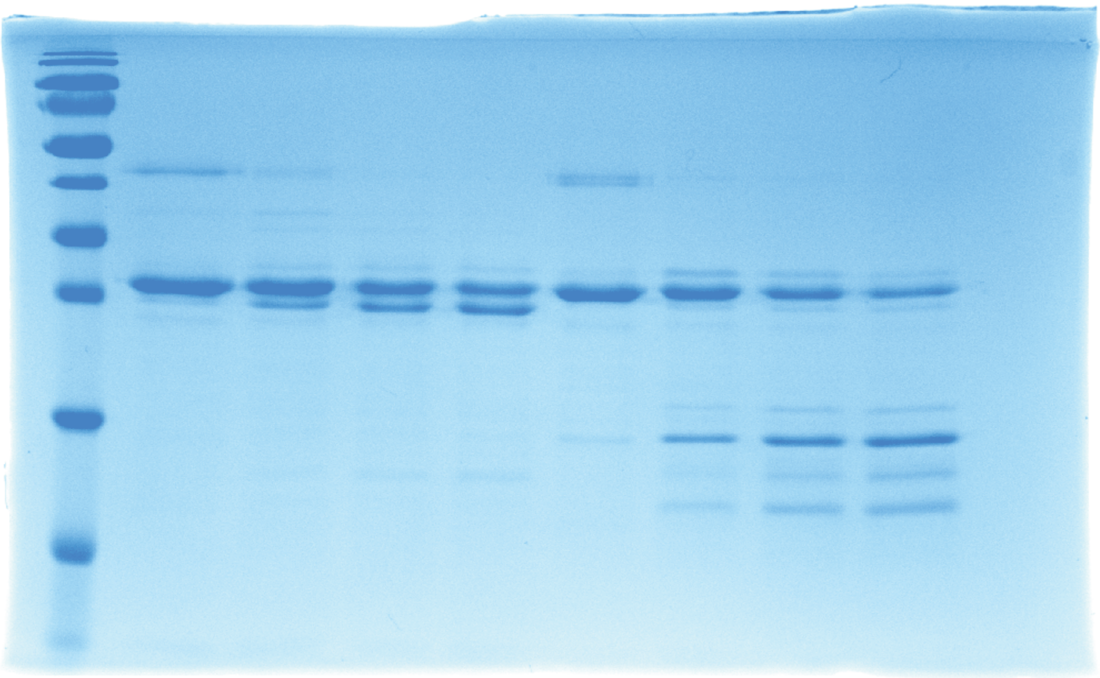

Figure 1 D and F

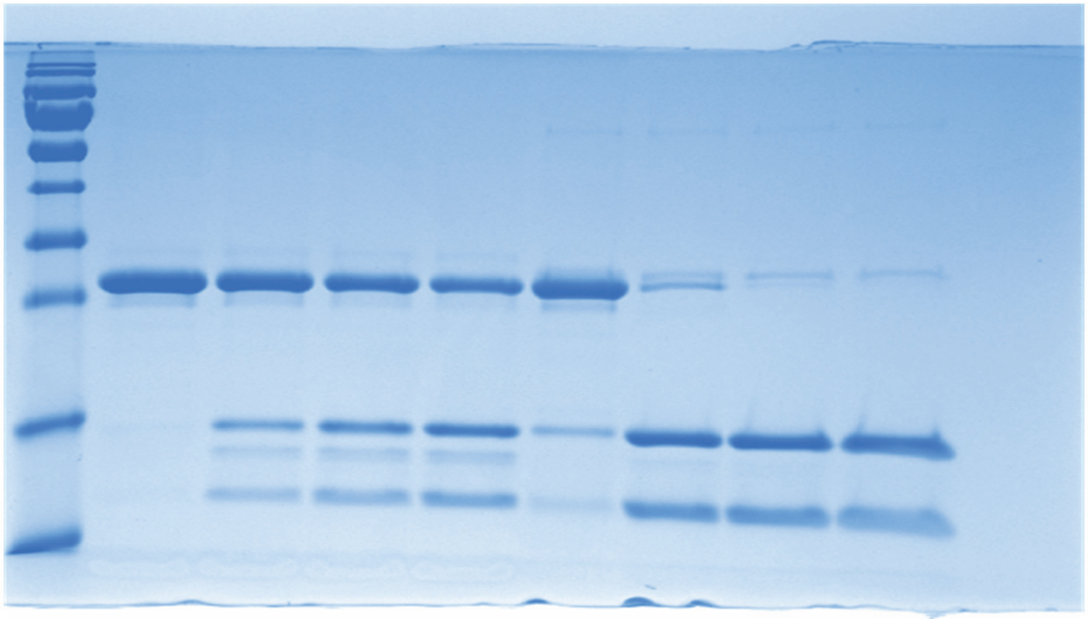

Supplementary Figure 4. Uncropped version of agarose gel shown in Figure 2C.

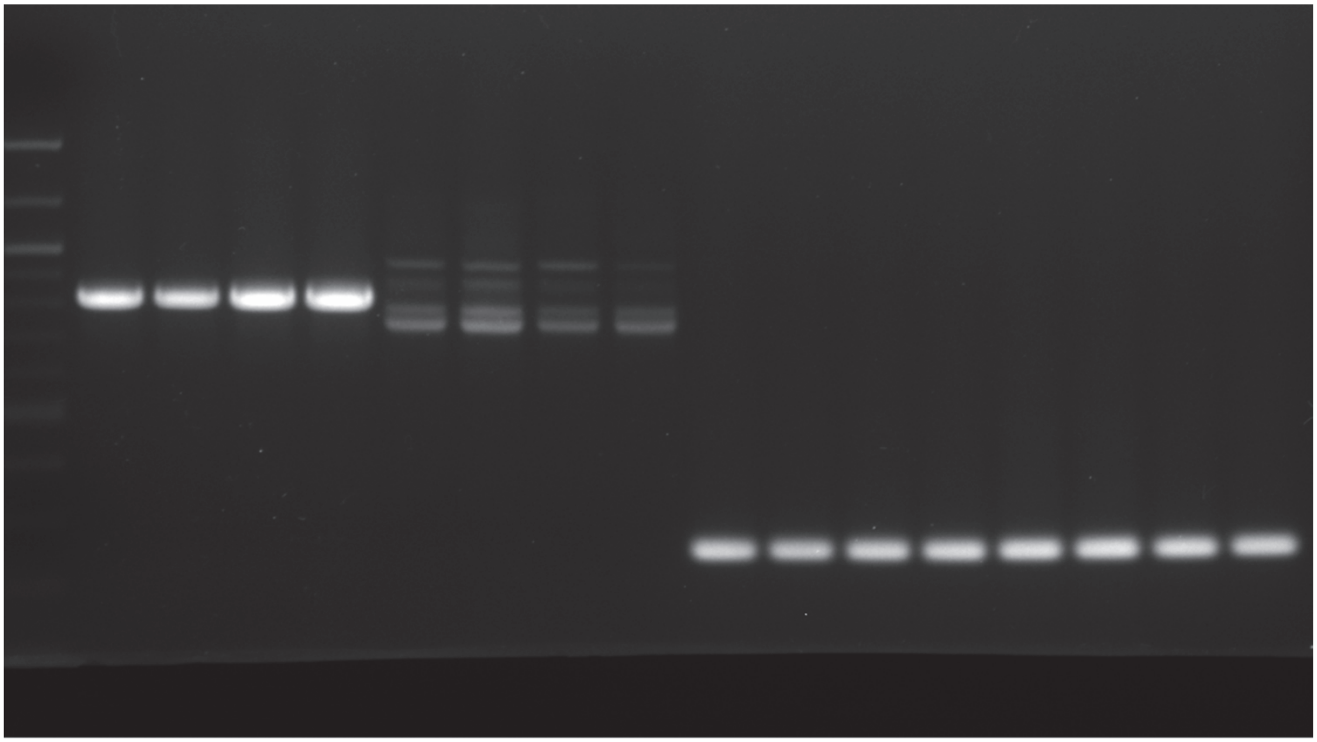

Supplementary Figure 5. Uncropped version of western blots shown in Figure 2D.

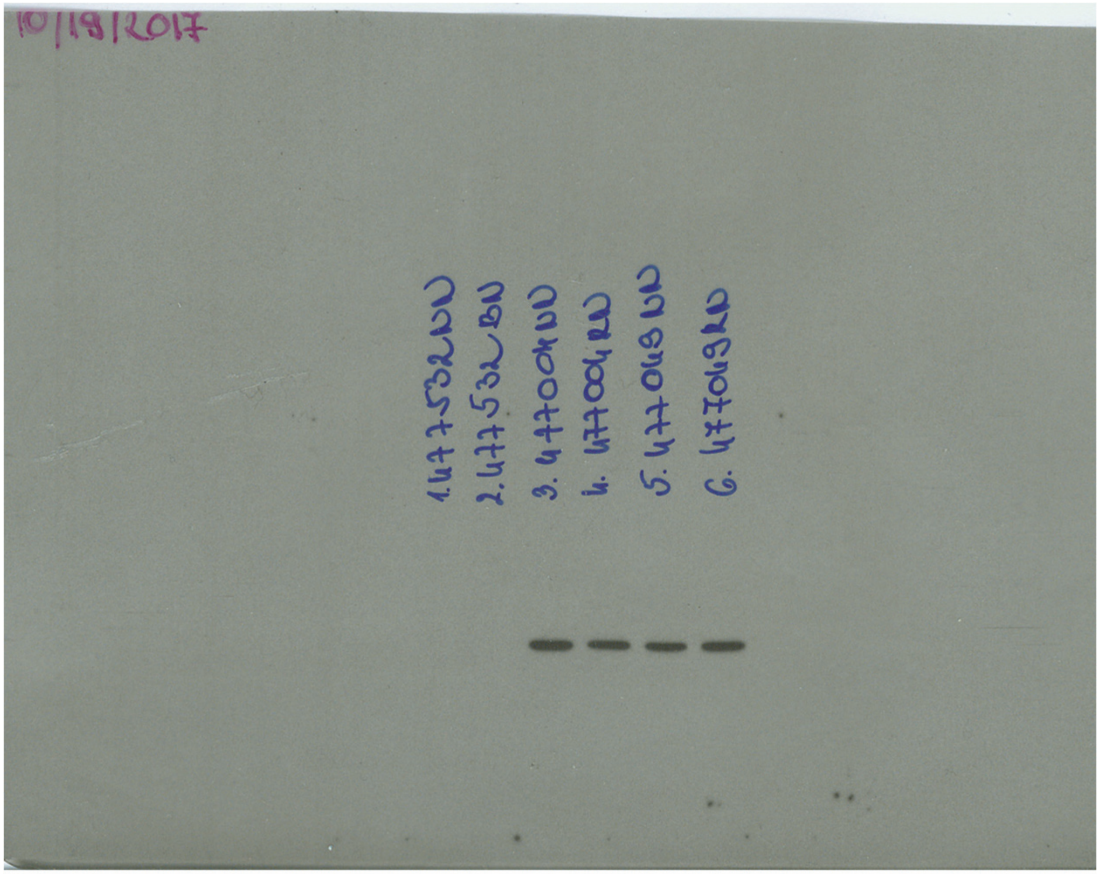

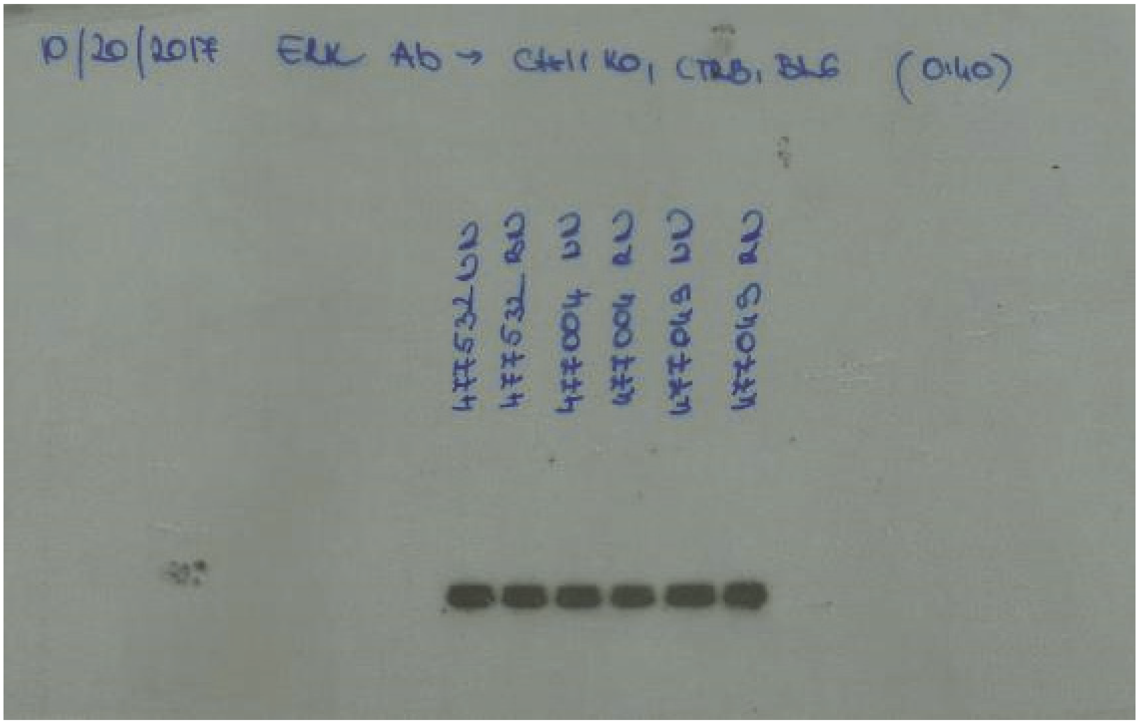

Supplement: Supplementary file 1 — Supplementary file1 (PDF 1778 kb) [file 41598_2020_68616_MOESM1_ESM.pdf]
